# The Cajal Body Protein WRAP53β Prepares the Scene for Repair of DNA Double-Strand Breaks by Regulating Local Ubiquitination

**DOI:** 10.3389/fmolb.2019.00051

**Published:** 2019-07-04

**Authors:** Sofie Bergstrand, Eleanor M. O'Brien, Marianne Farnebo

**Affiliations:** ^1^Department of Biosciences and Nutrition, Karolinska Institutet, Stockholm, Sweden; ^2^Department of Cell and Molecular Biology, Karolinska Institutet, Stockholm, Sweden

**Keywords:** DNA repair, ubiquitin, WRAP53β, Cajal body, WD40, cancer, chromatin modification, RNF8

## Abstract

Proper repair of DNA double-strand breaks is critical for maintaining genome integrity and avoiding disease. Modification of damaged chromatin has profound consequences for the initial signaling and regulation of repair. One such modification involves ubiquitination by E3 ligases RNF8 and RNF168 within minutes after DNA double-strand break formation, altering chromatin structure and recruiting factors such as 53BP1 and BRCA1 for repair via non-homologous end-joining (NHEJ) and homologous recombination (HR), respectively. The WD40 protein WRAP53β plays an essential role in localizing RNF8 to DNA breaks by scaffolding its interaction with the upstream factor MDC1. Loss of WRAP53β impairs ubiquitination at DNA lesions and reduces downstream repair by both NHEJ and HR. Intriguingly, WRAP53β depletion attenuates repair of DNA double-strand breaks more than depletion of RNF8, indicating functions other than RNF8-mediated ubiquitination. WRAP53β plays key roles with respect to the nuclear organelles Cajal bodies, including organizing the genome to promote associated transcription and collecting factors involved in maturation of the spliceosome and telomere elongation within these organelles. It is possible that similar functions may aid also in DNA repair. Here we describe the involvement of WRAP53β in Cajal bodies and DNA double-strand break repair in detail and explore whether and how these processes may be linked. We also discuss the possibility that the overexpression of WRAP53β detected in several cancer types may reflect its normal participation in the DNA damage response rather than oncogenic properties.

## The Lead Role for WRAP53β: Scaffolding Rna-Protein Complexes

### Function Through Organization

Structural organization within the nuclear space contributes significantly to functional regulation (Misteli, 2005; Nunez et al., 2009; Van Bortle and Corces, 2012). For example, organizing appropriate ribonucleoprotein complexes into the nuclear organelles known as Cajal bodies controls and accelerates reactions involved in pre-mRNA splicing and telomere elongation (Carmo-Fonseca et al., 1992; Jády et al., 2004; Kiss et al., 2006; Matera and Shpargel, 2006; Stanek and Neugebauer, 2006). Similarly, upon DNA damage, repair factors are concentrated into foci, providing an environment beneficial for repair (Bekker-Jensen et al., 2006; Altmeyer et al., 2015).

Cajal bodies contain transcription factors and polymerases (Polak et al., 2003; Machyna et al., 2013; Hutten et al., 2014) and like other nuclear bodies, can be formed in association with transcription (Shevtsov and Dundr, 2011). For Cajal bodies, this nucleation occurs at specific genomic loci, including genes encoding small nuclear (sn)RNAs, small nucleolar (sno)RNAs, small Cajal body-specific (sca)RNAs and histones (Frey and Matera, 1995, 2001; Smith et al., 1995; Machyna et al., 2013). When transcribed, these loci are brought together in a transcriptional center within the Cajal body that accelerates RNA production (Sawyer et al., 2016a,b; Wang et al., 2016). The RNAs transcribed are subsequently processed, modified (methylated, pseudouridinylated) and/or function within the Cajal bodies themselves (Darzacq et al., 2002; Jády et al., 2003; Dominski and Marzluff, 2007; Enwerem et al., 2015). Similarly, RNAs are transcribed from sites of DNA damage (Francia et al., 2012; Wei et al., 2012; Michelini et al., 2017; Bonath et al., 2018), which can hybridize with the damaged DNA (Ohle et al., 2016; Lu et al., 2018), be processed by DICER and DROSHA (Francia et al., 2012; Michelini et al., 2017; Lu et al., 2018) or become methylated by METTL3 (Xiang et al., 2017), thereafter, regulating damage repair.

### The Genome and Cajal Bodies Come Together With WRAP53β

**Figure 1:**
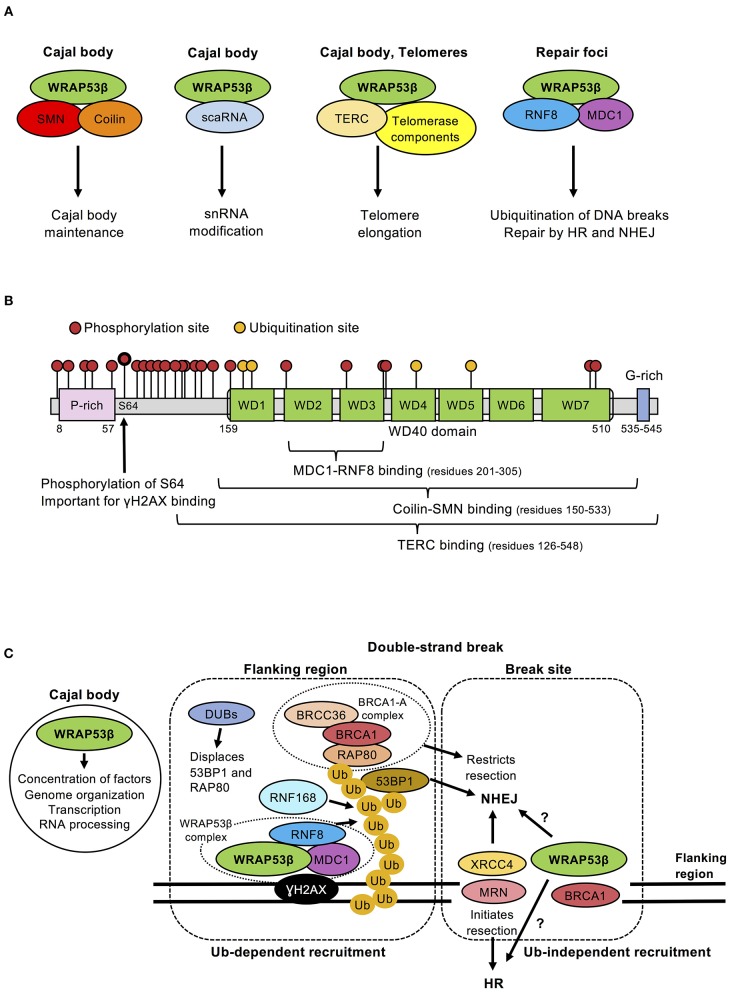
**(A)** Schematic illustration of the different WRAP53β complexes, their localization and function. Note: scaRNA and TERC are RNA molecules. **(B)** Schematic illustration of the domains, binding, phosphorylation and ubiquitination sites in the WRAP53β protein. The sites for post-translational modifications were obtained from PhosphoSitePlus on April 17, 2019. The location of WD40 repeats were predicted using the WD40-repeat protein Structures Predictor (Wu et al., 2012; Wang et al., 2013, 2015; Ma et al., 2019); (WDSP, May 2nd 2019): WD40 1 (amino acid residues 159–197), WD40 2 (residues 207–259), WD40 3 (residues 266–305), WD40 4 (residues 313–354), WD40 5 (residues 358–397), WD40 6 (residues 402–442), WD40 7 (residues 450–510). **(C)** Schematic view of the functions of WRAP53β in Cajal bodies, at the break site and in surrounding chromatin. Ubiquitin-dependent recruitment of DNA repair factors occurs at regions flanking the break site. WRAP53β binds γH2AX and also scaffolds the interaction between MDC1 and RNF8, which is important for the recruitment of RNF8 to DNA breaks. Once there, RNF8 and RNF168 ubiquitinate proteins at damaged chromatin, which stimulates recruitment of downstream factors 53BP1, RAD51, and BRCA1. BRCA1 forms several sub-complexes with different functions, of which the BRCA1-A complex (containing BRCA1, RAP80, BRCC36, and additional proteins not discussed here) restrict resection. Recruitment to the break site appears to be ubiquitin-independent and the factors recruited here include XRCC4, which promotes NHEJ, or DNA break sensor proteins, such as the MRN complex that promote HR. Pools of WRAP53β and BRCA1 also locate at this site for reasons unknown. Functions performed by WRAP53β in Cajal bodies could potentially be performed at break sites. The recruitment of RAD51, a downstream protein of WRAP53β, to DNA lesions appears to occur via both ubiquitin-dependent and independent mechanisms.

The scaffold protein WRAP53β (WD40-encoding RNA antisense to p53) (alias WRAP53, WDR79, and TCAB1), initially discovered in our laboratory as an antisense gene to p53 (Mahmoudi et al., 2009), plays several key roles in Cajal bodies. First, this protein is vital for their formation (Mahmoudi et al., 2010), bringing the necessary proteins and gene loci into close proximity (Mahmoudi et al., 2010; Wang et al., 2016). Loss of WRAP53β disrupts Cajal bodies, suppresses clustering of sn/sno/scaRNA/histone loci and downregulates transcription from these sites. Second, WRAP53β plays essential roles in maintaining Cajal bodies and targeting factors to these organelles (Figure 1A; Mahmoudi et al., 2010; Henriksson and Farnebo, 2015), probably by stabilizing interactions between Cajal body components. The direct interaction between the Cajal body marker Coilin and the splicing-related survival of motor neuron (SMN) protein is stabilized by WRAP53β (Mahmoudi et al., 2010), which can bind several proteins and RNAs simultaneously through its seven WD40 repeats (Figures 1A,B). WRAP53β also binds the telomerase RNA (TERC) and locates the telomerase complex to Cajal bodies and further on to telomeres (Venteicher et al., 2009).

Extensively interacting chromosomal loci are often fragile and enriched in DNA repair factors, indicating that they are primed for rapid DNA repair (Sobhy et al., 2019). WRAP53β is involved in DNA repair and its presence at Cajal body-associated gene loci may thus allow rapid repair. In addition, assembly of WRAP53β at DNA lesions may facilitate damage-induced genome reorganization and/or clustering of DNA breaks, which promotes efficient recognition and repair of lesions (Aymard et al., 2017; Stadler and Richly, 2017). WRAP53β may also stimulate transcription of RNA from the break site and/or its processing or concentrate repair factors into specialized foci to accelerate necessary reactions.

## Spotlight on Dna Repair: WRAP53β Controls Local Ubiquitination

### Regulation of Protein Recruitment and Repair Pathway Choice by Ubiquitination

Among the most toxic DNA lesions are double-strand breaks, repaired either by non-homologous end joining (NHEJ) or homologous recombination (HR) (reviewed e.g., by Ciccia and Elledge, 2010; Ceccaldi et al., 2016). Following such breakage, the damaged chromatin is modified chemically, including by ubiquitination, which facilitates recruitment of repair factors. Ubiquitination involves stepwise enzymatic activation, conjugation, and ligation of the small ubiquitin protein to lysine residues by E1, E2, and E3 enzymes, respectively (Pickart and Eddins, 2004). The presence of lysine residues in ubiquitin itself allows formation of various types of polyubiquitin chains, with ubiquitin chains linked at K48 typically targeting proteins for degradation, whereas K63-linked chains often signal protein recruitment (Panier and Durocher, 2009; Smeenk and van Attikum, 2013).

RNF8, the first ubiquitin ligase to arrive at DNA breaks (Huen et al., 2007; Kolas et al., 2007; Mailand et al., 2007), initially catalyzes K63-linked ubiquitin chains on histone H1 (Thorslund et al., 2015), which recruits RNF168 to ubiquitinate histone H2A at K13/K15 (Mattiroli et al., 2012; Uckelmann and Sixma, 2017), thereby potentiating the local ubiquitin signal (Uckelmann and Sixma, 2017). This triggers recruitment of 53BP1 (via K15 ubiquitination of H2A), which then restricts DNA end resection and promotes NHEJ repair (Figure 1C; Nakamura et al., 2006; Kolas et al., 2007; Fradet-Turcotte et al., 2013). Intriguingly, ubiquitin chains also recruit RAP80 along with the key HR factor BRCA1 (Sobhian et al., 2007; Wang and Elledge, 2007).

The choice of repair pathway beyond this point remains unknown. In addition to the known determinants [i.e., cell cycle phase, site of damage (e.g., gene-rich/poor regions) and local concentration of factors required], the BRCA1 recruited to ubiquitin as part of the BRCA1-A complex appears to be involved in fine-tuning the choice of repair pathway, since this complex attenuates end resection (Figure 1C; Sobhian et al., 2007; Wang and Elledge, 2007; Hu et al., 2011). In contrast, BRCA1 recruited via resected DNA belonging to other complexes (i.e., BRCA1-B, BRCA1-C, BRCA1-D) promotes end resection, strand invasion and RAD51 loading, crucial steps in the HR pathway (Greenberg et al., 2006; Sy et al., 2009; Zhang et al., 2009; Xie et al., 2012; Cruz-García et al., 2014; Savage and Harkin, 2015; Zhao et al., 2017). Moreover, BRCA1 has E3 ligase activity and, with the E3 ligase BARD1, can ubiquitinate H2A to remove 53BP1, thereby promoting HR (Densham et al., 2016).

Other ubiquitin ligases also promote HR and influence NHEJ by fine-tuning ubiquitination of damaged chromatin. For example RNF20 and RNF40 ubiquitinate histone H2B (Moyal et al., 2011; Nakamura et al., 2011; So et al., 2019), while UBE4A edits ubiquitin chains at breaks (Baranes-Bachar et al., 2018).

Deubiquitinating enzymes (DUBs) are also involved in the choice of repair pathway. Thus, removal of ubiquitin chains by POH1 promotes HR by displacing 53BP1 and RAP80 to the periphery of the repair foci (Butler et al., 2012; Kakarougkas et al., 2013; Nakada, 2016). Some BRCA1 complexes contain DUBs, including BRCC36, which functions together with the BRCA1-A complex; removal of this DUB results in unrestrained end resection and hyperactive HR (Figure 1C; Shao et al., 2009; Ng et al., 2016).

These observations emphasize the central role of ubiquitination in the DNA damage response, in which the RNF8/RNF168-pathway is a key upstream actor, regulating several steps of both NHEJ and HR repair. Notably, the RNF8 protein is unstable, so continuous splicing is required for its presence at DNA lesions (Pederiva et al., 2016). Consequently, even short term inhibition of splicing impairs repair (Pederiva et al., 2016), which can explain the defective repair associated with knockdown of various splicing factors detected in several genome-wide siRNA screens (Paulsen et al., 2009; Adamson et al., 2012).

### Alteration of Chromatin Structure by Ubiquitination

The structure of chromatin around DNA lesions influences the DNA damage response. Initial compaction stimulates early steps in this process, such as recruitment of the MRN complex, while persistent compaction is unfavorable to downstream repair and recovery, and attenuates phosphorylation of CHK2 (Burgess et al., 2014). Moreover, a collar of compact chromatin is formed around the DNA lesions, potentially to restrict repair to this site, since repair factors, including 53BP1, only localize within its decompacted interior (Lou et al., 2019).

Interestingly, this compaction around the break site is dependent on RNF8 (Lou et al., 2019), indicating a role for ubiquitination in regulating the higher-order structure of damaged chromatin. Since RNF8 can promote relaxation of chromatin by recruiting the remodeling factor CHD4 (Luijsterburg et al., 2012), it is possible that interior decondensation by RNF8 triggers the formation of a heterochromatic border around DNA breaks. Altogether, the ubiquitin response not only stimulates recruitment of repair factors and influences the choice of DNA double-strand break repair pathway but also appears to shape the local chromatin for proper progression of the DNA damage response.

### WRAP53β Orchestrates Ubiquitination of Damaged Chromatin via RNF8

WRAP53β was first implicated in DNA repair by several screens for novel repair proteins (Matsuoka et al., 2007; Paulsen et al., 2009; Adamson et al., 2012). Its direct involvement in the repair of DNA double-strand breaks by both HR and NHEJ was later confirmed and shown to involve scaffolding interactions between RNF8 and MDC1 (Henriksson et al., 2014) by simultaneously and independently binding the FHA domains of both proteins through its own WD40 domain (Figure 1B; Henriksson et al., 2014). In this manner, WRAP53β promotes assembly of RNF8 at DNA lesions, ubiquitination of damaged chromatin and downstream recruitment of 53BP1, BRCA1, and RAD51 (Figure 1A; Henriksson et al., 2014; Hedström et al., 2015). RNF8 and MDC1 can interact directly, but do not in the absence of WRAP53β, which appears to stabilize their interaction in a manner similar to the SMN-coilin interaction (Figure 1A). WRAP53β does not influences RNF8 levels, excluding indirect effects on splicing.

## Behind the Scenes: WRAP53β Plays Multiple Roles at Dna Breaks

### WRAP53β Influences DNA Repair Beyond RNF8

Notably, depletion of WRAP53β reduces HR and NHEJ efficiency more than knockdown of RNF8 (Henriksson et al., 2014), indicating that WRAP53β plays additional roles, probably ubiquitin-independent, in DNA repair. Indeed, two distinct WRAP53β fractions are present at DNA double-strand breaks; one in regions surrounding the break (also positive for γH2AX/MDC1/RNF8) and another at the break site itself [normally devoid of/low in γH2AX/MDC1/RNF8 (Henriksson et al., 2014; Goldstein and Kastan, 2015), but instead enriched in NHEJ factors (e.g., XRCC4) and DNA break sensors (e.g., NBS1, part of the MRN complex) (Goldstein et al., 2013)]. WRAP53β is recruited to the break site itself more rapidly and remains there longer than in the surrounding regions (Figure 1C; Henriksson et al., 2014).

Interestingly, like WRAP53β, BRCA1 is recruited to both regions and to a higher extent to the break site. Its recruitment to the surrounding regions depends on the RNF8/RNF168/RAP80-pathway (and on ATM and PARP), while its accumulation at the break site is mediated by the MRN complex. BRCA1 appears to stimulate cell cycle checkpoints at the flanking regions and re-ligation of the breaks at the break site (Xu et al., 2001; Goldstein and Kastan, 2015). Thus, WRAP53β and BRCA1 both participate in the RNF8-mediated ubiquitination pathway, while promoting other aspects of repair at the break site itself (Figure 1C).

### What Regulates WRAP53β?

Recruitment of WRAP53β to repair foci, probably the regions surrounding DNA double-strand breaks, requires ATM, H2AX and MDC1 (Henriksson et al., 2014). Importantly, upon DNA damage, WRAP53β is phosphorylated by ATM at serine 64 (Matsuoka et al., 2007; Coucoravas et al., 2017) and a phosphomutant of WRAP53β (S64A) cannot rescue defects in DNA repair when the wild-type protein is knocked down (Rassoolzadeh et al., 2015; Coucoravas et al., 2017). ATM-mediated phosphorylation of WRAP53β does not influence its interaction with RNF8 and MDC1. However, WRAP53β also binds γH2AX and this interaction is enhanced by phosphorylation (Figure 1B; Rassoolzadeh et al., 2015; Coucoravas et al., 2017). Since WRAP53β binds RNF8 and MDC1 even before damage, these three proteins might pre-form a complex that can be activated and recruited to DNA breaks by ATM in a multistep manner, e.g., phosphorylation of MDC1 allows direct RNF8-MDC1 interaction, phosphorylation of WRAP53β stimulates WRAP53β-γH2AX interaction and phosphorylation of γH2AX allows MDC1-γH2AX interaction (Rassoolzadeh et al., 2015; Coucoravas et al., 2017).

Phosphorylated WRAP53β^S64^ locates to both DNA breaks and Cajal bodies. However, the unphosphorylated form accumulates in Cajal bodies to a greater extent (Coucoravas et al., 2017), indicating that phosphorylation of WRAP53β by ATM relocates this protein from Cajal bodies to DNA breaks. WRAP53β targets several factors to Cajal bodies and maintains the structure of this organelle and these functions may be affected by WRAP53β relocation. For example, ionizing radiation moves WRAP53β to DNA breaks, while telomerase (Wong et al., 2002) and several other Cajal body components (including coilin, SMN, fibrillarin, and snRNAs) move to and around nucleoli (in nucleolar caps). This indicates that exit of WRAP53β from Cajal bodies displaces associated proteins to other sites. Moreover, Cajal bodies become disrupted several hours after DNA damage (Gilder et al., 2011).

In addition to S64, 23 other residues of WRAP53β are phosphorylated and four ubiquitinated by unknown enzymes for unclear reason (Figure 1B) (data from UniProt and PhosphoSitePlus websites) (Hebert and Poole, 2017). WRAP53β appears to be rate-limiting for both HR and NHEJ and, accordingly, its overexpression enhances the efficiency of both pathways by stimulating RNF8-mediated ubiquitination at damaged chromatin (Rassoolzadeh et al., 2016). Further studies on the complex interplay between the functions of WRAP53β in DNA repair, the Cajal body and telomere maintenance are required and post-translational modifications may be important in this context.

## WRAP53β Acting Off-Script: Loss of Tumor Suppression and Activation of the Dna Damage Response In Cancer

### Loss of Tumor Suppression by WRAP53β

**Figure 2:**
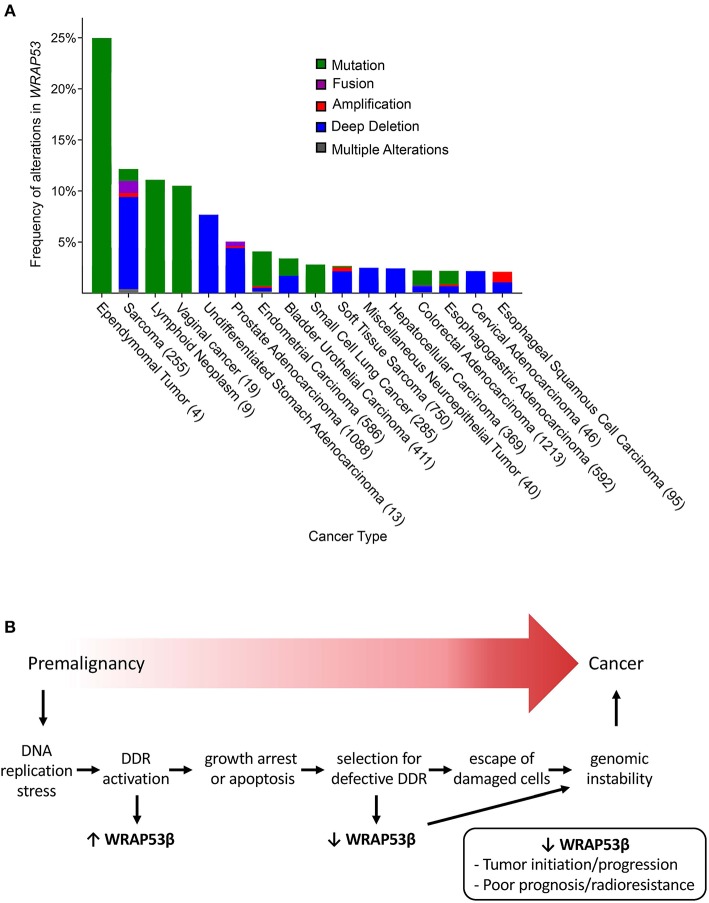
**(A)** The frequency of genetic alterations in *WRAP53* associated with various cancer types (minimum threshold of 2%) (From cBioPortal, accessed April 17, 2019). The numbers in parenthesis represent the number of patients analyzed. **(B)** Proposed model for the involvement of WRAP53β and the DNA damage response in the development of cancer. Aberrant cell proliferation may cause replication stress, formation of DNA double-strand breaks and activation of the DNA damage response. Overexpression of WRAP53β as part of the response stimulates repair, growth arrest and/or apoptosis, but some damaged cells may escape (e.g., due to downregulation of WRAP53β), leading to genomic instability and potential progression into cancer.

Inactivating germline mutations in WRAP53β cause dyskeratosis congenita, characterized by bone marrow failure, premature aging and predisposition for cancer (Zhong et al., 2011). Moreover, downregulation of WRAP53β RNA or its loss from the nucleus in patients with head and neck, breast, and ovarian cancer is correlated with shorter survival (Garvin et al., 2015; Hedström et al., 2015; Silwal-Pandit et al., 2015). Furthermore, numerous genetic alterations in *WRAP53*, mainly deletions or mutations, are present in multiple cancers (Figure 2A) (cBioPortal, https://www.cbioportal.org/) (Cerami et al., 2012; Gao et al., 2013), further evidence that loss-of-WRAP53β-function promotes cancer development/progression. In addition, attenuated expression of WRAP53β correlates with resistance of patients with head and neck cancer and metastasized rectal cancer to radiotherapy (Zhang et al., 2012; Garvin et al., 2015).

At the same time, WRAP53β is overexpressed in other cancer types (see further below), but the clinical relevance remains unclear. Although we have suggested that WRAP53β has oncogenic properties (Mahmoudi et al., 2011), we now believe that this is a misinterpretation of the data, which instead reflects participation of WRAP53β in the DNA damage response (Figure 2B).

### Does WRAP53β Appear to be Oncogenic by Activating the DNA Damage Response?

Precancerous lesions are characterized by activation of DNA damage signaling and repair, often due to replication stress, which is believed to constrain tumor progression. This phenomenon includes formation of 53BP1 foci and activation of the ATM/ATR checkpoint; premalignant tumor samples stain positively for the phosphorylated forms of ATM, CHK1, CHK2, RAD17, p53, and H2AX (Bartkova et al., 2005; Gorgoulis et al., 2005). Potential inactivating mutations in key DNA damage response proteins, such as p53 and ATM (Olivier et al., 2010; Choi et al., 2016) allow survival of damaged and genetically unstable cells that upon clonal expansion progress into carcinoma (Bartkova et al., 2005; Gorgoulis et al., 2005).

WRAP53β is overexpressed in a variety of cancer cell lines and primary head and neck, lung and colorectal cancers (Zhang et al., 2012; Rao et al., 2014; Sun et al., 2014, 2016; Zhu et al., 2018). In addition, knockdown of this protein promotes apoptosis and reduces proliferation of cancer cell lines and xenografts (Mahmoudi et al., 2011; Sun et al., 2014, 2016; Wang et al., 2017; Yuan et al., 2017; Chen et al., 2018; Zhu et al., 2018). Such observations appeared to indicate that WRAP53β may act as an oncogene. However, in the vast majority of studies to date overexpression of WRAP53β was not significantly associated with worse patient survival.

Instead, overexpression of WRAP53β may reflect its involvement in DNA repair and thus be a response to the stress of rapid proliferation. Further support for this proposal includes the following: (1) upregulation of WRAP53β promotes activation of the ATR-CHK1 pathway in nasopharyngeal carcinoma induced by Epstein Barr Virus (Wang et al., 2017); (2) upregulation of WRAP53β correlates with activation of the DNA damage response pathway in ovarian cancer (Hedström et al., 2015); (3) subsequent downregulation of WRAP53β in patients with ovarian cancer is significantly associated with higher mortality, while intitial upregulation was not, indicating that downregulation drives tumor progression (Hedström et al., 2015); and (4) similarly, downregulation of WRAP53β in patients with metastasized rectal cancer promotes resistance to radiotherapy and is associated with higher mortality, while initial upregulation did not influence patient survival (Zhang et al., 2012). Moreover, enhanced expression of WRAP53β in cancer cells may be linked to their greater number of Cajal bodies, reflecting a higher demand for associated functions (Spector et al., 1992).

Potential re-activation of telomerase is, as far as we can see, the only reasonable mechanism by which upregulation of WRAP53β could actually promote tumorigenesis. However, the telomerase gene is not an oncogene, since its product does not by itself cause uncontrolled growth and is active in normal embryonic stem and germline cells (Harley, 2002). Several studies have reported a correlation between overexpression of WRAP53β and increased telomere length or telomerase activity, but it remains to be determined whether telomerase activity is correlated with prognosis (Qiu et al., 2015; Wang et al., 2017; Sun et al., 2018).

## Concluding Remarks—Wrapping it all up

The scaffolding protein WRAP53β organizes the genome so that formation of Cajal bodies is stimulated, the expression of associated genes enhanced and their products concentrated in these organelles. Similarly, WRAP53β concentrates factors important for repair of DNA double-strand breaks via ubiquitination of damaged chromatin. Upon DNA damage, a fraction of WRAP53β is phosphorylated, promoting its role in DNA repair, with several other pools of WRAP53β having different localizations and functions, orchestrated by various post-translational modifications. This complexity explains how this protein performs so many tasks within the cell in a coordinated fashion, as well as why disease may occur when it is lost or dysfunctional.

In addition to dyskeratosis congenita and sporadic cancer, loss of WRAP53β has been linked to the pathogenesis of spinal muscular atrophy (Mahmoudi et al., 2010; Di Giorgio et al., 2017). Furthermore, this protein is part of a repair machinery that organizes and resolves persistent DNA damage in neurons (Mata-Garrido et al., 2016) and accumulation of such damage is believed to contribute to neurodegenerative disorders such as spinal muscular atrophy and amyotrophic lateral sclerosis (Fayzullina and Martin, 2014; Mitra et al., 2019).

Thus, more in-depth understanding of the role of WRAP53β in DNA repair and other processes may help decipher the complicated mechanisms underlying tumorigenesis, premature aging and neurodegeneration and thereby lead to novel treatment strategies.

## Author Contributions

SB, EO, and MF wrote the manuscript, were involved in the generation of the figures and critically revised the manuscript. All authors contributed to the conception of this review article and MF co-ordinated the work.

### Conflict of Interest Statement

The authors declare that the research was conducted in the absence of any commercial or financial relationships that could be construed as a potential conflict of interest.

## Abbreviations

53BP1: p53-binding protein 1
ATM: Ataxia-telangiectasia mutated
ATR: Ataxia telangiectasia and Rad3 related
BARD1: BRCA1-associated RING domain protein 1
BRCA1: Breast cancer type 1 susceptibility protein
BRCC36: BRCA1/BRCA2-containing complex subunit 36
CHD4: Chromodomain-helicase-DNA-binding protein 4
CHK1: Checkpoint kinase 1
CHK2: Checkpoint kinase 2
DUB: Deubiquitinating enzyme
FHA domain: Forkhead-associated domain
H1: Histone 1
H2A: Histone 2A
H2B: Histone 2B
H2AX: Histone variant 2AX
γH2AX: Phosphorylated histone H2AX
HR: Homologous recombination
MDC1: mediator of DNA damage checkpoint 1
METTL3: N6-adenosine-methyltransferase 70 kDa subunit
MRN, Complex with MRE11 homolog double strand break repair nuclease: RAD50 double strand break repair protein and Nibrin
NBS1: Nibrin
NHEJ: Non-homologous end joining
PARP: Poly (ADP-ribose) polymerase
POH1: proteasome 19S subunit
RAD17: DNA repair protein RAD17 homolog
RAD51: DNA repair protein RAD51 homolog
RAP80: Receptor-associated protein 80
RNF8: Ring finger protein 8
RNF20: Ring finger protein 20
RNF40: Ring finger protein 40
RNF168: Ring finger protein 168
scaRNA: Small Cajal body-specific RNA
SMN: Survival of motor neuron protein
snRNA: Small nuclear RNA
snoRNA: Small nucleolar RNA
UBE4A: Ubiquitin conjugation factor E4 A
WRAP53β: WD40-encoding RNA antisense to p53
XRCC4: X-ray repair cross-complementing protein 4.

## References

[B1] Adamson B. Smogorzewska A. Sigoillot F. D. King R. W. Elledge S. J. (2012). A genome-wide homologous recombination screen identifies the RNA-binding protein RBMX as a component of the DNA-damage response. Nat. Cell Biol. 14, 318–328. 10.1038/ncb2426 22344029 PMC3290715

[B2] Altmeyer M. Neelsen K. J. Teloni F. Pozdnyakova I. Pellegrino S. Grøfte M.. (2015). Liquid demixing of intrinsically disordered proteins is seeded by poly(ADP-ribose). Nat. Commun. 6:8088. 10.1038/ncomms9088 26286827 PMC4560800

[B3] Aymard F. Aguirrebengoa M. Guillou E. Javierre B. M. Bugler B. Arnould C.. (2017). Genome-wide mapping of long-range contacts unveils clustering of DNA double-strand breaks at damaged active genes. Nat. Struct. Mol. Biol. 24, 353–361. 10.1038/nsmb.3387 28263325 PMC5385132

[B4] Baranes-Bachar K. Levy-Barda A. Oehler J. Reid D. A. Soria-Bretones I. Voss T. C.. (2018). The ubiquitin E3/E4 ligase UBE4A adjusts protein ubiquitylation and accumulation at sites of DNA damage, facilitating double-strand break repair. Mol. Cell 69, 866–878.e867. 10.1016/j.molcel.2018.02.002 29499138 PMC6265044

[B5] Bartkova J. Horejsí Z. Koed K. Krämer A. Tort F. Zieger K.. (2005). DNA damage response as a candidate anti-cancer barrier in early human tumorigenesis. Nature 434, 864–870. 10.1038/nature03482 15829956

[B6] Bekker-Jensen S. Lukas C. Kitagawa R. Melander F. Kastan M. B. Bartek J.. (2006). Spatial organization of the mammalian genome surveillance machinery in response to DNA strand breaks. J. Cell Biol. 173, 195–206. 10.1083/jcb.200510130 16618811 PMC2063811

[B7] Bonath F. Domingo-Prim J. Tarbier M. Friedländer M. R. Visa N. (2018). Next-generation sequencing reveals two populations of damage-induced small RNAs at endogenous DNA double-strand breaks. Nucleic Acids Res. 46, 11869–11882. 10.1093/nar/gky1107 30418607 PMC6294500

[B8] Burgess R. C. Burman B. Kruhlak M. J. Misteli T. (2014). Activation of DNA damage response signaling by condensed chromatin. Cell Rep. 9, 1703–1717. 10.1016/j.celrep.2014.10.060 25464843 PMC4267891

[B9] Butler L. R. Densham R. M. Jia J. Garvin A. J. Stone H. R. Shah V.. (2012). The proteasomal de-ubiquitinating enzyme POH1 promotes the double-strand DNA break response. EMBO J. 31, 3918–3934. 10.1038/emboj.2012.232 22909820 PMC3463844

[B10] Carmo-Fonseca M. Pepperkok R. Carvalho M. T. Lamond A. I. (1992). Transcription-dependent colocalization of the U1, U2, U4/U6, and U5 snRNPs in coiled bodies. J. Cell Biol. 117, 1–14. 10.1083/jcb.117.1.1 1532583 PMC2289407

[B11] Ceccaldi R. Rondinelli B. D'Andrea A. D. (2016). Repair pathway choices and consequences at the double-strand break. Trends Cell Biol. 26, 52–64. 10.1016/j.tcb.2015.07.009 26437586 PMC4862604

[B12] Cerami E. Gao J. Dogrusoz U. Gross B. E. Sumer S. O. Aksoy B. A.. (2012). The cBio cancer genomics portal: an open platform for exploring multidimensional cancer genomics data. Cancer Discov. 2, 401–404. 10.1158/2159-8290.CD-12-0095 22588877 PMC3956037

[B13] Chen J. Sheng X. Ma H. Tang Z. Yang C. Cao L.. (2018). WDR79 mediates the proliferation of non-small cell lung cancer cells by regulating the stability of UHRF1. J. Cell. Mol. Med. 22, 2856–2864. 10.1111/jcmm.13580 29516630 PMC5908104

[B14] Choi M. Kipps T. Kurzrock R. (2016). ATM mutations in cancer: therapeutic implications. Mol. Cancer Ther. 15, 1781–1791. 10.1158/1535-7163.MCT-15-0945 27413114

[B15] Ciccia A. Elledge S. J. (2010). The DNA damage response: making it safe to play with knives. Mol. Cell 40, 179–204. 10.1016/j.molcel.2010.09.019 20965415 PMC2988877

[B16] Coucoravas C. Dhanjal S. Henriksson S. Böhm S. Farnebo M. (2017). Phosphorylation of the Cajal body protein WRAP53beta by ATM promotes its involvement in the DNA damage response. RNA Biol. 14, 804–813. 10.1080/15476286.2016.1243647 27715493 PMC5519231

[B17] Cruz-García A. López-Saavedra A. Huertas P. (2014). BRCA1 accelerates CtIP-mediated DNA-end resection. Cell Rep. 9, 451–459. 10.1016/j.celrep.2014.08.076 25310973

[B18] Darzacq X. Jády B. E. Verheggen C. Kiss A. M. Bertrand E. Kiss T. (2002). Cajal body-specific small nuclear RNAs: a novel class of 2′-O-methylation and pseudouridylation guide RNAs. EMBO J. 21, 2746–2756. 10.1093/emboj/21.11.2746 12032087 PMC126017

[B19] Densham R. M. Garvin A. J. Stone H. R. Strachan J. Baldock R. A. Daza-Martin M.. (2016). Human BRCA1-BARD1 ubiquitin ligase activity counteracts chromatin barriers to DNA resection. Nat. Struct. Mol. Biol. 23, 647–655. 10.1038/nsmb.3236 27239795 PMC6522385

[B20] Di Giorgio M. L. Esposito A. Maccallini P. Micheli E. Bavasso F. Gallotta I.. (2017). WDR79/TCAB1 plays a conserved role in the control of locomotion and ameliorates phenotypic defects in SMA models. Neurobiol. Dis. 105, 42–50. 10.1016/j.nbd.2017.05.005 28502804

[B21] Dominski Z. Marzluff W. F. (2007). Formation of the 3′ end of histone mRNA: getting closer to the end. Gene 396, 373–390. 10.1016/j.gene.2007.04.021 17531405 PMC2888136

[B22] Enwerem II Wu G. Yu Y. T. Hebert M. D. (2015). Cajal body proteins differentially affect the processing of box C/D scaRNPs. PLoS ONE 10:e0122348. 10.1371/journal.pone.0122348 25875178 PMC4395269

[B23] Fayzullina S. Martin L. J. (2014). Skeletal muscle DNA damage precedes spinal motor neuron DNA damage in a mouse model of Spinal Muscular Atrophy (SMA). PLoS ONE 9:e93329. 10.1371/journal.pone.0093329 24667816 PMC3965546

[B24] Fradet-Turcotte A. Canny M. D. Escribano-Díaz C. Orthwein A. Leung C. C. Huang H.. (2013). 53BP1 is a reader of the DNA-damage-induced H2A Lys 15 ubiquitin mark. Nature 499, 50–54. 10.1038/nature12318 23760478 PMC3955401

[B25] Francia S. Michelini F. Saxena A. Tang D. de Hoon M. Anelli V.. (2012). Site-specific DICER and DROSHA RNA products control the DNA-damage response. Nature 488, 231–235. 10.1038/nature11179 22722852 PMC3442236

[B26] Frey M. R. Matera A. G. (1995). Coiled bodies contain U7 small nuclear RNA and associate with specific DNA sequences in interphase human cells. Proc. Natl. Acad. Sci. U.S.A. 92, 5915–5919. 10.1073/pnas.92.13.5915 7597053 PMC41612

[B27] Frey M. R. Matera A. G. (2001). RNA-mediated interaction of Cajal bodies and U2 snRNA genes. J. Cell Biol. 154, 499–509. 10.1083/jcb.200105084 11489914 PMC2196410

[B28] Gao J. Aksoy B. A. Dogrusoz U. Dresdner G. Gross B. Sumer S. O.. (2013). Integrative analysis of complex cancer genomics and clinical profiles using the cBioPortal. Sci. Signal. 6:pl1. 10.1126/scisignal.2004088 23550210 PMC4160307

[B29] Garvin S. Tiefenböck K. Farnebo L. Thunell L. K. Farnebo M. Roberg K. (2015). Nuclear expression of WRAP53beta is associated with a positive response to radiotherapy and improved overall survival in patients with head and neck squamous cell carcinoma. Oral Oncol. 51, 24–30. 10.1016/j.oraloncology.2014.10.003 25456005

[B30] Gilder A. S. Do P. M. Carrero Z. I. Cosman A. M. Broome H. J. Velma V.. (2011). Coilin participates in the suppression of RNA polymerase I in response to cisplatin-induced DNA damage. Mol. Biol. Cell 22, 1070–1079. 10.1091/mbc.e10-08-0731 21289084 PMC3069010

[B31] Goldstein M. Derheimer F. A. Tait-Mulder J. Kastan M. B. (2013). Nucleolin mediates nucleosome disruption critical for DNA double-strand break repair. Proc. Natl. Acad. Sci. U.S.A. 110, 16874–16879. 10.1073/pnas.1306160110 24082117 PMC3801049

[B32] Goldstein M. Kastan M. B. (2015). Repair versus checkpoint functions of BRCA1 are differentially regulated by site of chromatin binding. Cancer Res. 75, 2699–2707. 10.1158/0008-5472.CAN-15-0400 25939603 PMC4548823

[B33] Gorgoulis V. G. Vassiliou L. V. Karakaidos P. Zacharatos P. Kotsinas A. Liloglou T.. (2005). Activation of the DNA damage checkpoint and genomic instability in human precancerous lesions. Nature 434, 907–913. 10.1038/nature03485 15829965

[B34] Greenberg R. A. Sobhian B. Pathania S. Cantor S. B. Nakatani Y. Livingston D. M. (2006). Multifactorial contributions to an acute DNA damage response by BRCA1/BARD1-containing complexes. Genes Dev. 20, 34–46. 10.1101/gad.1381306 16391231 PMC1356099

[B35] Harley C. B. (2002). Telomerase is not an oncogene. Oncogene 21, 494–502. 10.1038/sj.onc.1205076 11850774

[B36] Hebert M. D. Poole A. R. (2017). Towards an understanding of regulating Cajal body activity by protein modification. RNA Biol. 14, 761–778. 10.1080/15476286.2016.1243649 27819531 PMC5519237

[B37] Hedström E. Pederiva C. Farnebo J. Nodin B. Jirström K. Brennan D. J.. (2015). Downregulation of the cancer susceptibility protein WRAP53beta in epithelial ovarian cancer leads to defective DNA repair and poor clinical outcome. Cell Death Dis. 6:e1892. 10.1038/cddis.2015.250 26426684 PMC4632285

[B38] Henriksson S. Farnebo M. (2015). On the road with WRAP53beta: guardian of Cajal bodies and genome integrity. Front. Genet. 6:91. 10.3389/fgene.2015.00091 25852739 PMC4371746

[B39] Henriksson S. Rassoolzadeh H. Hedström E. Coucoravas C. Julner A. Goldstein M.. (2014). The scaffold protein WRAP53beta orchestrates the ubiquitin response critical for DNA double-strand break repair. Genes Dev. 28, 2726–2738. 10.1101/gad.246546.114 25512560 PMC4265676

[B40] Hu Y. Scully R. Sobhian B. Xie A. Shestakova E. Livingston D. M. (2011). RAP80-directed tuning of BRCA1 homologous recombination function at ionizing radiation-induced nuclear foci. Genes Dev. 25, 685–700. 10.1101/gad.2011011 21406551 PMC3070932

[B41] Huen M. S. Grant R. Manke I. Minn K. Yu X. Yaffe M. B.. (2007). RNF8 transduces the DNA-damage signal via histone ubiquitylation and checkpoint protein assembly. Cell 131, 901–914. 10.1016/j.cell.2007.09.041 18001825 PMC2149842

[B42] Hutten S. Chachami G. Winter U. Melchior F. Lamond A. I. (2014). A role for the Cajal-body-associated SUMO isopeptidase USPL1 in snRNA transcription mediated by RNA polymerase II. J. Cell Sci. 127(Pt 5), 1065–1078. 10.1242/jcs.141788 24413172 PMC3937775

[B43] Jády B. E. Bertrand E. Kiss T. (2004). Human telomerase RNA and box H/ACA scaRNAs share a common Cajal body-specific localization signal. J. Cell Biol. 164, 647–652. 10.1083/jcb.200310138 14981093 PMC2172171

[B44] Jády B. E. Darzacq X. Tucker K. E. Matera A. G. Bertrand E. Kiss T. (2003). Modification of Sm small nuclear RNAs occurs in the nucleoplasmic Cajal body following import from the cytoplasm. EMBO J. 22, 1878–1888. 10.1093/emboj/cdg187 12682020 PMC154478

[B45] Kakarougkas A. Ismail A. Katsuki Y. Freire R. Shibata A. Jeggo P. A. (2013). Co-operation of BRCA1 and POH1 relieves the barriers posed by 53BP1 and RAP80 to resection. Nucleic Acids Res. 41, 10298–10311. 10.1093/nar/gkt802 24013561 PMC3905848

[B46] Kiss T. Fayet E. Jády B. E. Richard P. Weber M. (2006). Biogenesis and intranuclear trafficking of human box C/D and H/ACA RNPs. Cold Spring Harb. Symp. Quant. Biol. 71, 407–417. 10.1101/sqb.2006.71.025 17381323

[B47] Kolas N. K. Chapman J. R. Nakada S. Ylanko J. Chahwan R. Sweeney F. D.. (2007). Orchestration of the DNA-damage response by the RNF8 ubiquitin ligase. Science 318, 1637–1640. 10.1126/science.1150034 18006705 PMC2430610

[B48] Lou J. Scipioni L. Wright B. K. Bartolec T. K. Zhang J. Masamsetti V. P.. (2019). Phasor histone FLIM-FRET microscopy quantifies spatiotemporal rearrangement of chromatin architecture during the DNA damage response. Proc. Natl. Acad. Sci. U.S.A. 116, 7323–7332. 10.1073/pnas.1814965116 30918123 PMC6462080

[B49] Lu W. T. Hawley B. R. Skalka G. L. Baldock R. A. Smith E. M. Bader A. S.. (2018). Drosha drives the formation of DNA:RNA hybrids around DNA break sites to facilitate DNA repair. Nat. Commun. 9:532. 10.1038/s41467-018-02893-x 29416038 PMC5803274

[B50] Luijsterburg M. S. Acs K. Ackermann L. Wiegant W. W. Bekker-Jensen S. Larsen D. H.. (2012). A new non-catalytic role for ubiquitin ligase RNF8 in unfolding higher-order chromatin structure. EMBO J. 31, 2511–2527. 10.1038/emboj.2012.104 22531782 PMC3365417

[B51] Ma J. An K. Zhou J. B. Wu N. S. Wang Y. Ye Z. Q.. (2019). WDSPdb: an updated resource for WD40 proteins. Bioinformatics 10.1093/bioinformatics/btz460. [Epub ahead of print]. 31161214 PMC6853709

[B52] Machyna M. Heyn P. Neugebauer K. M. (2013). Cajal bodies: where form meets function. Wiley Interdiscipl. Rev. RNA 4, 17–34. 10.1002/wrna.1139 23042601

[B53] Mahmoudi S. Henriksson S. Corcoran M. Méndez-Vidal C. Wiman K. G. Farnebo M. (2009). Wrap53, a natural p53 antisense transcript required for p53 induction upon DNA damage. Mol. Cell 33, 462–471. 10.1016/j.molcel.2009.01.028 19250907

[B54] Mahmoudi S. Henriksson S. Farnebo L. Roberg K. Farnebo M. (2011). WRAP53 promotes cancer cell survival and is a potential target for cancer therapy. Cell Death Dis. 2:e114. 10.1038/cddis.2010.90 21368886 PMC3077286

[B55] Mahmoudi S. Henriksson S. Weibrecht I. Smith S. Söderberg O. Strömblad S.. (2010). WRAP53 is essential for Cajal body formation and for targeting the survival of motor neuron complex to Cajal bodies. PLoS Biol. 8:e1000521. 10.1371/journal.pbio.1000521 21072240 PMC2970535

[B56] Mailand N. Bekker-Jensen S. Faustrup H. Melander F. Bartek J. Lukas C.. (2007). RNF8 ubiquitylates histones at DNA double-strand breaks and promotes assembly of repair proteins. Cell 131, 887–900. 10.1016/j.cell.2007.09.040 18001824

[B57] Mata-Garrido J. Casafont I. Tapia O. Berciano M. T. Lafarga M. (2016). Neuronal accumulation of unrepaired DNA in a novel specific chromatin domain: structural, molecular and transcriptional characterization. Acta Neuropathol. Commun. 4:41. 10.1186/s40478-016-0312-9 27102221 PMC4840862

[B58] Matera A. G. Shpargel K. B. (2006). Pumping RNA: nuclear bodybuilding along the RNP pipeline. Curr. Opin. Cell Biol. 18, 317–324. 10.1016/j.ceb.2006.03.005 16632338

[B59] Matsuoka S. Ballif B. A. Smogorzewska A. McDonald E. R. 3rd, Hurov, K. E. Luo J.. (2007). ATM and ATR substrate analysis reveals extensive protein networks responsive to DNA damage. Science 316, 1160–1166. 10.1126/science.1140321 17525332

[B60] Mattiroli F. Vissers J. H. van Dijk W. J. Ikpa P. Citterio E. Vermeulen W.. (2012). RNF168 ubiquitinates K13-15 on H2A/H2AX to drive DNA damage signaling. Cell 150, 1182–1195. 10.1016/j.cell.2012.08.005 22980979

[B61] Michelini F. Pitchiaya S. Vitelli V. Sharma S. Gioia U. Pessina F.. (2017). Damage-induced lncRNAs control the DNA damage response through interaction with DDRNAs at individual double-strand breaks. Nat. Cell Biol. 19, 1400–1411. 10.1038/ncb3643 29180822 PMC5714282

[B62] Misteli T. (2005). Concepts in nuclear architecture. BioEssays. 27, 477–487. 10.1002/bies.20226 15832379

[B63] Mitra J. Guerrero E. N. Hegde P. M. Liachko N. F. Wang H. Vasquez V. (2019). Motor neuron disease-associated loss of nuclear TDP-43 is linked to DNA double-strand break repair defects. Proc. Natl. Acad. Sci. U.S.A. 116, 4696–4705. 10.1073/pnas.1818415116 PMC6410842 30770445

[B64] Moyal L. Lerenthal Y. Gana-Weisz M. Mass G. So S. Wang S. Y.. (2011). Requirement of ATM-dependent monoubiquitylation of histone H2B for timely repair of DNA double-strand breaks. Mol. Cell 41, 529–542. 10.1016/j.molcel.2011.02.015 21362549 PMC3397146

[B65] Nakada S. (2016). Opposing roles of RNF8/RNF168 and deubiquitinating enzymes in ubiquitination-dependent DNA double-strand break response signaling and DNA-repair pathway choice. J. Radiat. Res. 57(Suppl 1), i33–i40. 10.1093/jrr/rrw027 26983989 PMC4990112

[B66] Nakamura K. Kato A. Kobayashi J. Yanagihara H. Sakamoto S. Oliveira D. V.. (2011). Regulation of homologous recombination by RNF20-dependent H2B ubiquitination. Mol. Cell 41, 515–528. 10.1016/j.molcel.2011.02.002 21362548

[B67] Nakamura K. Sakai W. Kawamoto T. Bree R. T. Lowndes N. F. Takeda S.. (2006). Genetic dissection of vertebrate 53BP1: a major role in non-homologous end joining of DNA double strand breaks. DNA Repair (Amst). 5, 741–749. 10.1016/j.dnarep.2006.03.008 16644291

[B68] Ng H. M. Wei L. Lan L. Huen M. S. (2016). The Lys63-deubiquitylating enzyme BRCC36 limits DNA break processing and repair. J. Biol. Chem. 291, 16197–16207. 10.1074/jbc.M116.731927 27288411 PMC4965568

[B69] Nunez E. Fu X. D. Rosenfeld M. G. (2009). Nuclear organization in the 3D space of the nucleus - cause or consequence? Curr. Opin. Genet. Dev. 19, 424–436. 10.1016/j.gde.2009.07.005 19846290 PMC2796509

[B70] Ohle C. Tesorero R. Schermann G. Dobrev N. Sinning I. Fischer T. (2016). Transient RNA-DNA hybrids are required for efficient double-strand break repair. Cell 167, 1001–1013.e1007. 10.1016/j.cell.2016.10.001 27881299

[B71] Olivier M. Hollstein M. Hainaut P. (2010). TP53 mutations in human cancers: origins, consequences, and clinical use. Cold Spring Harb. Perspect. Biol. 2:a001008. 10.1101/cshperspect.a001008 20182602 PMC2827900

[B72] Panier S. Durocher D. (2009). Regulatory ubiquitylation in response to DNA double-strand breaks. DNA Repair (Amst). 8, 436–443. 10.1016/j.dnarep.2009.01.013 19230794

[B73] Paulsen R. D. Soni D. V. Wollman R. Hahn A. T. Yee M. C. Guan A.. (2009). A genome-wide siRNA screen reveals diverse cellular processes and pathways that mediate genome stability. Mol. Cell 35, 228–239. 10.1016/j.molcel.2009.06.021 19647519 PMC2772893

[B74] Pederiva C. Böhm S. Julner A. Farnebo M. (2016). Splicing controls the ubiquitin response during DNA double-strand break repair. Cell Death Differ. 23, 1648–1657. 10.1038/cdd.2016.58 27315300 PMC5041194

[B75] Pickart C. M. Eddins M. J. (2004). Ubiquitin: structures, functions, mechanisms. Biochim. Biophys. Acta 1695, 55–72. 10.1016/j.bbamcr.2004.09.019 15571809

[B76] Polak P. E. Simone F. Kaberlein J. J. Luo R. T. Thirman M. J. (2003). ELL and EAF1 are Cajal body components that are disrupted in MLL-ELL leukemia. Mol. Biol. Cell 14, 1517–1528. 10.1091/mbc.e02-07-0394 12686606 PMC153119

[B77] Qiu H. Zhao D. Y. Yuan L. M. Zhang G. Xie C. H. (2015). Regulatory effects of WRAP53 on radiosensitivity of laryngeal squamous cell carcinoma cells. Asian Pac. J. Cancer Prev. 16, 2975–2979. 10.7314/APJCP.2015.16.7.2975 25854392

[B78] Rao X. Huang D. Sui X. Liu G. Song X. Xie J.. (2014). Overexpression of WRAP53 is associated with development and progression of esophageal squamous cell carcinoma. PLoS ONE 9:e91670. 10.1371/journal.pone.0091670 24626331 PMC3953598

[B79] Rassoolzadeh H. Böhm S. Hedström E. Gad H. Helleday T. Henriksson S.. (2016). Overexpression of the scaffold WD40 protein WRAP53beta enhances the repair of and cell survival from DNA double-strand breaks. Cell Death Dis. 7:e2267. 10.1038/cddis.2016.172 27310875 PMC5143398

[B80] Rassoolzadeh H. Coucoravas C. Farnebo M. (2015). The proximity ligation assay reveals that at DNA double-strand breaks WRAP53beta associates with gammaH2AX and controls interactions between RNF8 and MDC1. Nucleus 6, 417–424. 10.1080/19491034.2015.1106675 26734725 PMC4915514

[B81] Savage K. I. Harkin D. P. (2015). BRCA1, a ‘complex' protein involved in the maintenance of genomic stability. FEBS J. 282, 630–646. 10.1111/febs.13150 25400280

[B82] Sawyer I. A. Shevtsov S. P. Dundr M. (2016a). Spectral imaging to visualize higher-order genomic organization. Nucleus 7, 325–338. 10.1080/19491034.2016.1187344 27167405 PMC4991238

[B83] Sawyer I. A. Sturgill D. Sung M. H. Hager G. L. Dundr M. (2016b). Cajal body function in genome organization and transcriptome diversity. Bioessays 38, 1197–1208. 10.1002/bies.201600144 27767214 PMC5225948

[B84] Shao G. Lilli D. R. Patterson-Fortin J. Coleman K. A. Morrissey D. E. Greenberg R. A. (2009). The Rap80-BRCC36 de-ubiquitinating enzyme complex antagonizes RNF8-Ubc13-dependent ubiquitination events at DNA double strand breaks. Proc. Natl. Acad. Sci. U.S.A. 106, 3166–3171. 10.1073/pnas.0807485106 19202061 PMC2651241

[B85] Shevtsov S. P. Dundr M. (2011). Nucleation of nuclear bodies by RNA. Nat. Cell Biol. 13, 167–173. 10.1038/ncb2157 21240286

[B86] Silwal-Pandit L. Russnes H. Borgen E. Skarpeteig V. Moen Vollan H. K. Schlichting E.. (2015). The sub-cellular localization of WRAP53 has prognostic impact in breast cancer. PLoS ONE 10:e0139965. 10.1371/journal.pone.0139965 26460974 PMC4603798

[B87] Smeenk G. van Attikum H. (2013). The chromatin response to DNA breaks: leaving a mark on genome integrity. Annu. Rev. Biochem. 82, 55–80. 10.1146/annurev-biochem-061809-174504 23414304

[B88] Smith K. P. Carter K. C. Johnson C. V. Lawrence J. B. (1995). U2 and U1 snRNA gene loci associate with coiled bodies. J. Cell. Biochem. 59, 473–485. 10.1002/jcb.240590408 8749717

[B89] So C. C. Ramachandran S. Martin A. (2019). E3 ubiquitin ligases RNF20 and RNF40 are required for double-stranded break (DSB) repair: evidence for monoubiquitination of histone H2B lysine 120 as a novel axis of DSB signaling and repair. Mol. Cell. Biol. 39:e00488–18. 10.1128/MCB.00488-18 30692271 PMC6447412

[B90] Sobhian B. Shao G. Lilli D. R. Culhane A. C. Moreau L. A. Xia B.. (2007). RAP80 targets BRCA1 to specific ubiquitin structures at DNA damage sites. Science 316, 1198–1202. 10.1126/science.1139516 17525341 PMC2706583

[B91] Sobhy H. Kumar R. Lewerentz J. Lizana L. Stenberg P. (2019). Highly interacting regions of the human genome are enriched with enhancers and bound by DNA repair proteins. Sci. Rep. 9:4577. 10.1038/s41598-019-40770-9 30872630 PMC6418152

[B92] Spector D. L. Lark G. Huang S. (1992). Differences in snRNP localization between transformed and nontransformed cells. Mol. Biol. Cell 3, 555–569. 10.1091/mbc.3.5.555 1535243 PMC275608

[B93] Stadler J. Richly H. (2017). Regulation of DNA repair mechanisms: how the chromatin environment regulates the DNA damage response. Int. J. Mol. Sci. 18:E1715. 10.3390/ijms18081715 28783053 PMC5578105

[B94] Stanek D. Neugebauer K. M. (2006). The Cajal body: a meeting place for spliceosomal snRNPs in the nuclear maze. Chromosoma 115, 343–354. 10.1007/s00412-006-0056-6 16575476

[B95] Sun C. K. Luo X. B. Gou Y. P. Hu L. Wang K. Li C.. (2014). TCAB1: a potential target for diagnosis and therapy of head and neck carcinomas. Mol. Cancer 13:180. 10.1186/1476-4598-13-180 25070141 PMC4118648

[B96] Sun H. Kim P. Jia P. Park A. K. Liang H. Zhao Z. (2018). Distinct telomere length and molecular signatures in seminoma and non-seminoma of testicular germ cell tumor. Brief Bioinform. 10.1093/bib/bby020. [Epub ahead of print]. 29579225 PMC6781582

[B97] Sun Y. Yang C. Chen J. Song X. Li Z. Duan M.. (2016). Overexpression of WDR79 in non-small cell lung cancer is linked to tumour progression. J. Cell. Mol. Med. 20, 698–709. 10.1111/jcmm.12759 26849396 PMC5125931

[B98] Sy S. M. Huen M. S. Chen J. (2009). PALB2 is an integral component of the BRCA complex required for homologous recombination repair. Proc. Natl. Acad. Sci. U.S.A. 106, 7155–7160. 10.1073/pnas.0811159106 19369211 PMC2678481

[B99] Thorslund T. Ripplinger A. Hoffmann S. Wild T. Uckelmann M. Villumsen B.. (2015). Histone H1 couples initiation and amplification of ubiquitin signalling after DNA damage. Nature 527, 389–393. 10.1038/nature15401 26503038

[B100] Uckelmann M. Sixma T. K. (2017). Histone ubiquitination in the DNA damage response. DNA Repair (Amst). 56, 92–101. 10.1016/j.dnarep.2017.06.011 28624371

[B101] Van Bortle K. Corces V. G. (2012). Nuclear organization and genome function. Annu. Rev. Cell Dev. Biol. 28, 163–187. 10.1146/annurev-cellbio-101011-155824 22905954 PMC3717390

[B102] Venteicher A. S. Abreu E. B. Meng Z. McCann K. E. Terns R. M. Veenstra T. D.. (2009). A human telomerase holoenzyme protein required for Cajal body localization and telomere synthesis. Science 323, 644–648. 10.1126/science.1165357 19179534 PMC2728071

[B103] Wang B. Elledge S. J. (2007). Ubc13/Rnf8 ubiquitin ligases control foci formation of the Rap80/Abraxas/Brca1/Brcc36 complex in response to DNA damage. Proc. Natl. Acad. Sci. U.S.A. 104, 20759–20763. 10.1073/pnas.0710061104 18077395 PMC2410075

[B104] Wang K. Ge Y. Ni C. Cui B. Du J. Zhang B.. (2017). Epstein-Barr virus-induced up-regulation of TCAB1 is involved in the DNA damage response in nasopharyngeal carcinoma. Sci. Rep. 7:3218. 10.1038/s41598-017-03156-3 28607398 PMC5468285

[B105] Wang Q. Sawyer I. A. Sung M. H. Sturgill D. Shevtsov S. P. Pegoraro G.. (2016). Cajal bodies are linked to genome conformation. Nat. Commun. 7:10966. 10.1038/ncomms10966 26997247 PMC4802181

[B106] Wang Y. Hu X. J. Zou X. D. Wu X. H. Ye Z. Q. Wu Y. D. (2015). WDSPdb: a database for WD40-repeat proteins. Nucleic Acids Res. 43, D339–344. 10.1093/nar/gku1023 25348404 PMC4383882

[B107] Wang Y. Jiang F. Zhuo Z. Wu X. H. Wu Y. D. (2013). A method for WD40 repeat detection and secondary structure prediction. PLoS ONE 8:e65705. 10.1371/journal.pone.0065705 23776530 PMC3679165

[B108] Wei W. Ba Z. Gao M. Wu Y. Ma Y. Amiard S.. (2012). A role for small RNAs in DNA double-strand break repair. Cell 149, 101–112. 10.1016/j.cell.2012.03.002 22445173

[B109] Wong J. M. Kusdra L. Collins K. (2002). Subnuclear shuttling of human telomerase induced by transformation and DNA damage. Nat. Cell Biol. 4, 731–736. 10.1038/ncb846 12198499

[B110] Wu X. H. Wang Y. Zhuo Z. Jiang F. Wu Y. D. (2012). Identifying the hotspots on the top faces of WD40-repeat proteins from their primary sequences by beta-bulges and DHSW tetrads. PLoS ONE 7:e43005. 10.1371/journal.pone.0043005 22916195 PMC3419727

[B111] Xiang Y. Laurent B. Hsu C. H. Nachtergaele S. Lu Z. Sheng W. (2017). RNA m(6)A methylation regulates the ultraviolet-induced DNA damage response. Nature 543, 573–576. 10.1038/nature21671 28297716 PMC5490984

[B112] Xie J. Peng M. Guillemette S. Quan S. Maniatis S. Wu Y.. (2012). FANCJ/BACH1 acetylation at lysine 1249 regulates the DNA damage response. PLoS Genet. 8:e1002786. 10.1371/journal.pgen.1002786 22792074 PMC3390368

[B113] Xu B. Kim S. Kastan M. B. (2001). Involvement of Brca1 in S-phase and G(2)-phase checkpoints after ionizing irradiation. Mol. Cell. Biol. 21, 3445–3450. 10.1128/MCB.21.10.3445-3450.2001 11313470 PMC100266

[B114] Yuan X. S. Cao L. X. Hu Y. J. Bao F. C. Wang Z. T. Cao J. L.. (2017). Clinical, cellular, and bioinformatic analyses reveal involvement of WRAP53 overexpression in carcinogenesis of lung adenocarcinoma. Tumour Biol. 39:1010428317694309. 10.1177/1010428317694309 28347242

[B115] Zhang F. Ma J. Wu J. Ye L. Cai H. Xia B.. (2009). PALB2 links BRCA1 and BRCA2 in the DNA-damage response. Curr. Biol. 19, 524–529. 10.1016/j.cub.2009.02.018 19268590 PMC2750839

[B116] Zhang H. Wang D. W. Adell G. Sun X. F. (2012). WRAP53 is an independent prognostic factor in rectal cancer- a study of Swedish clinical trial of preoperative radiotherapy in rectal cancer patients. BMC Cancer 12:294. 10.1186/1471-2407-12-294 22805008 PMC3504514

[B117] Zhao W. Steinfeld J. B. Liang F. Chen X. Maranon D. G. Jian Ma C.. (2017). BRCA1-BARD1 promotes RAD51-mediated homologous DNA pairing. Nature 550, 360–365. 10.1038/nature24060 28976962 PMC5800781

[B118] Zhong F. Savage S. A. Shkreli M. Giri N. Jessop L. Myers T.. (2011). Disruption of telomerase trafficking by TCAB1 mutation causes dyskeratosis congenita. Genes Dev. 25, 11–16. 10.1101/gad.2006411 21205863 PMC3012932

[B119] Zhu Y. Ding L. Chen B. F. Song J. G. Yao Y. S. (2018). Oncogenic activity of Wrap53 in human colorectal cancer *in vitro* and in nude mouse xenografts. Med. Sci. Monit. 24, 6129–6136. 10.12659/MSM.910214 30175821 PMC6131976

